# Medical Students' Reflections on Transitioning to Their First General Practice Placement: Qualitative Descriptive Study

**DOI:** 10.2196/76599

**Published:** 2026-03-12

**Authors:** Sara Bashar Qasrawi, Maryam Tomerak, Shahad Abdulkhaleq Mamalchi, Fatima Atieh, Hasan AlAdraj, Mohamed Abdulla, Salim Fredericks, Ghufran Jassim, Hani Malik, Eric Clarke, Denis Harkin, Shaista Salman Guraya

**Affiliations:** 1 School of Medicine, Royal College of Surgeons in Ireland - Medical University of Bahrain, Busaiteen, Bahrain; 2 Department of Surgery, University of Toronto, Toronto, ON, Canada; 3 Cardiff and Vale University Health Board, NHS, Cardiff, United Kingdom; 4 Salmaniya Medical Complex, Manama, Bahrain; 5 Family Medicine Department, School of Medicine, Royal College of Surgeons in Ireland (RCSI)-Medical University of Bahrain, Busaiteen, Bahrain; 6 Royal Bahrain Hospital, Manama, Bahrain; 7 Faculty of Medicine and Health Sciences, Royal College of Surgeons in Ireland, Dublin, Ireland; 8 Institute of Learning, Mohammed Bin Rashid University of Medicine and Health Sciences, Dubai Health, Al Razi St - Umm Hurair 2 - Dubai Healthcare City, Dubai, 505055, United Arab Emirates, 971 507073240

**Keywords:** medical education, professional identity formation, reflective writing, clinical learning environment, qualitative descriptive methodology

## Abstract

**Background:**

Transitioning from preclinical to clinical training is a critical milestone of “becoming and being” in a medical student’s journey. Despite simulation-based learning, real-world clinical exposure remains indispensable in shaping professional identity. The clinical learning environment is a complex interplay of social, cultural, and organizational factors that influence students’ development as future health care professionals.

**Objective:**

This study explores medical students’ reflections on their first clinical placement in general practice, aiming to understand their experiences, challenges, and the clinical learning environment’s role in their learning and developing professional identity formation as a step toward establishing a conceptual framework and a common language for educators, which we hope will promote further advances to support beneficial professional identity formation.

**Methods:**

We analyzed reflections from fourth-year medical students following their initial general practice placement. A qualitative descriptive approach grounded in naturalism was employed to explain our participants’ transitioning encounters in clear, everyday language to ensure their experiences were presented in their own words, without bias. Content thematic analysis was conducted to identify key themes related to their experiences.

**Results:**

Students’ reflections revealed a startled cohort unprepared for the epistemological, emotional, and practical realities of clinical work. Many assumed that classroom “knowing” would seamlessly translate into clinical “doing” but were met instead with uncertainty, failure, and emotional overwhelm, often manifesting as shame, guilt, and withdrawal. These experiences illuminated a fracture between knowledge and knowing, underscored by students’ prereflective epistemological beliefs and varying degrees of supervisory preparedness. While emotional and cognitive struggles were widespread, rare instances of supportive mentorship and feedback significantly bolstered students’ confidence and participation.

**Conclusions:**

Reflection offered a valuable window into how students think, feel, and act during this critical transition, but it is not a cure-all. Reflection should be positioned as a developmental and communal tool within a broader scaffolding that includes early skills preparation, role clarity, psychological safety, and trained supervisors. Structured shared reflective circles and narrative listening sessions can help normalize uncertainty, support identity formation, and foster resilience during students’ entry into clinical practice and can ease the journey of “becoming and being.”

## Introduction

Despite advances in simulation-based learning, the clinical learning environment (CLE) remains irreplaceable in health care education. Its unpredictable, ethically complex, and emotionally charged nature uniquely shapes how medical students think, feel, and act [1] as future health care professionals. As Nordquist et al [2] describe, the CLE is a dynamic interplay of social, cultural, organizational, and material factors in which students learn while working. Within this setting, students engage in situated learning [3], navigating the multifaceted realities of becoming a physician [4]. An effective CLE therefore influences learners’ well-being, engagement, and professional socialization [2].

The theory of legitimate peripheral participation (LPP) by Lave and Wenger [5] helps us understand how novices begin on the periphery of practice and, through increasingly authentic participation, move toward fuller membership in the medical community. These early encounters profoundly influence students’ confidence, role interpretation, and internalization of professional norms [6]. They integrate the values, attitudes, and ethical responsibilities of the profession into their evolving sense of self as they move from peripheral to central participation. Yet this movement is rarely linear; it is marked by dissonance, confusion, and emotional complexity requiring thoughtful processing. Psychological approaches [7] emphasize harnessing these moments through structured reflections, which can help them make sense of their experiences by charting their path within the profession.

Reflective writing has become a key pedagogical tool for helping learners articulate and process complex experiences [8]. By cultivating metacognitive and meta-affective capacities, reflective writing enables a personal voice infused with emotions, which helps individuals identify and rationalize their developing sense of self. Wald [7] used the metaphorical notion of a *resiliency workout* for guided and structured reflective writing, enabling medical students to regulate emotions, process feedback, and remain empathetically engaged with patients and colleagues. Primarily, in the early stages of clinical exposure, reflective writing can provide a structured pedagogical space for students to pause, process, and internalize the meaning of their experiences, providing them with a feeling of not only clinical competence but also practical wisdom, ethical clarity, and emotional resilience [9]. While reflection has long been recognized as essential to professional development, its theoretical underpinnings, particularly those rooted in epistemologies of practice and critical social inquiry, remain underexplored in medical education [10]. We echo recent calls for continued theorization of reflection, not only as a tool for self-regulation but also as a means to navigate uncertainty, challenge dominant discourses, and construct knowledge within complex clinical realities [11].

To illustrate this, examining how medical students themselves narrate and reflect on their early clinical experiences through the lens of LPP contributes meaningfully to ongoing scholarship of “becoming” and “being”—professional identity formation (PIF). A more descriptive-interpretive understanding allows medical educators to identify opportunities to support students’ PIF and facilitate their transition from preclinical learning to the CLE. In this research, we aimed to explore how medical students perceive and experience LPP in the CLE and how it influences their developing professional identity, as a step toward establishing a conceptual framework and a common language for educators, which we hope will promote further advances to support beneficial PIF.

## Methods

### Study Design

We employed a qualitative descriptive (QD) approach to address our research question. As described by Sandelowski [12], QD is grounded in naturalism, requiring researchers to stay close to the data and portray participants’ perceptions and experiences in their own words in an unadorned manner without introducing bias. The QD approach helped us explain the transitioning experiences of our participants using straightforward language. During data analysis, all involved researchers established their constructionist paradigmatic stance and acknowledged their sensitivities and inclinations. The research team engaged in reflexive discussions to remain aware of their own assumptions and positionalities, ensuring that interpretations remain grounded in participants’ narratives. The senior author, SSG, is an expert in medical education and medical professionalism, with higher degree qualifications and research publications. Coauthors SAM, HA, and MA were medical students and classmates of the participants. SBQ, MT, and FA were junior medical students at the Royal College of Surgeons in Ireland – Medical University of Bahrain (RCSI-MUB).

### Participant Recruitment

Naturalistic inquiry nudges researchers to ensure that the collected data are good enough to enable participants to express their experiences authentically and without external influence. Hence, we analyzed reflective essay accounts written by a cohort of fourth-year medical students during their first general practice (GP) placement to document the transition phase from preclinical learning to the CLE. As part of the assessment, these structured essays comprised 500‐700 words and were submitted using a “Plagiarism Checker,” Turnitin. Students were instructed to reflect according to the 6 stages (description, feelings, evaluation, analysis, conclusion, and action plan) of Gibbs’ reflective cycle [13]. We prompted students to document specific learning moments and challenges encountered during their first clinical placement. “Challenging or difficult” clinical encounters were considered to highlight the choices and narratives of such critical incidents, which reflect medical students’ values and attitudes in the context of professional experiences [14].

**Figure 1.**
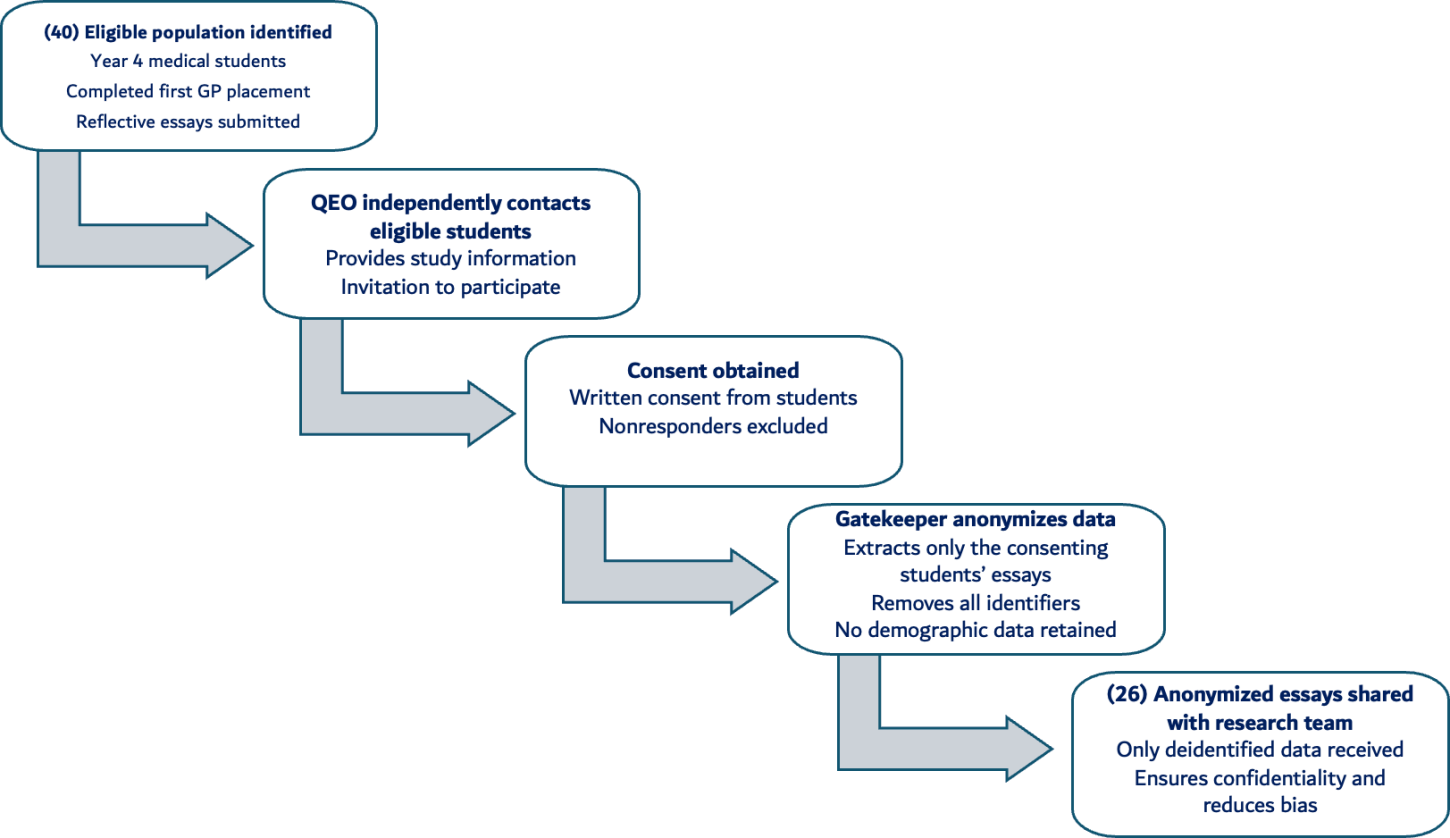
A flowchart outlining the recruitment and data management procedures for the study. GP: general practice; QEO: Quality Enhancement Office.

The study aimed to explore how students began visualizing themselves as part of the clinical team, capturing the transformations that occurred during this pivotal stage of their PIF. To answer our study’s objectives, we selected students who were attending their first clinical GP placement to ensure they had no significant prior exposure to clinical practice. To provide context to our readers, it is worth mentioning a brief overview of the RCSI-MUB curriculum. The first two and a half preclinical years are entirely theoretical and focus on basic science instruction. Clinical exposure begins in the second semester of year 3, and the first GP rotation occurs in year 4 (Figure 1).

### Ethical Considerations

Ethical approval was obtained from the RCSI-MUB Research Ethics Committee (REC/2023/153). Participant recruitment followed the flowchart process: the Quality Enhancement Office contacted a cohort of year 4 students who had completed their first GP placement and provided them with a study information sheet outlining the purpose of this study and the voluntary nature of participation. Written informed consent was obtained prior to inclusion. Participation or refusal had no academic or professional consequences, and no financial or academic compensation was provided. Only anonymized reflective essays from consenting students were shared with the research team. The gatekeeper removed all identifying and demographic information before sharing the essays, and the investigators had no access to participant identifiers. Data were stored securely on institutional systems accessible only to the research team.

### Context of Study

Students, before starting their GP placements, receive little advance notice that the CLE will challenge their assumptions. While they are introduced to the educational aspects of the program, including learning outcomes and assessment requirements, they receive minimal orientation to the realities of clinical practice. Clinical skills are introduced in the second half of year 3 in simulation and class-based settings, without any supervised or structured opportunities to practice on peers or real patients. Students are not adequately prepared for the structural, social, cultural, and practical complexities of patient interactions. Although elements of professionalism such as cultural competence, confidentiality, and informed consent are discussed, these remain largely theoretical and disconnected from clinical context.

Clinical placements occur at varied sites under the supervision of adjunct clinical faculty with differing levels of teaching expertise, a dilemma familiar to many medical schools without their own clinical facilities. GP tutors are expected to facilitate learning toward defined outcomes but receive no formal preparation in creating psychological safety, dealing with uncertainties, supervising students, debriefing, or providing structured feedback. As a result, much of their teaching relies on personal experience rather than standardized educational practice. Consequently, there are varying degrees of preparedness and engagement on both sides—students and tutors alike.

### Data Collection and Analysis

As common in QD, we used content thematic analysis [15,16] to identify and describe core themes across all data. First, SSG, SBQ, MT, SAM, and FA independently analyzed the first 6 essays to identify initial codes. Next, SAM, SBQ, MT, and FA consolidated the initial codes into subthemes and preliminary themes. All codes, coding schemas, notes, and memos were captured on Microsoft Excel, and version control was maintained throughout to ensure a transparent process. Later, SSG examined the worked Microsoft Excel spreadsheet and preliminary results and data with a naturalistic inquiry paradigm and envisioned that transitioning moments are marked with significant changes in thinking, feeling, and enacting like a certain individual. Using the flexibility of QD methodology to describe and interpret data based on the study’s purpose, we finalized interpretive themes, which were then applied to the remaining essays. Finally, the results were vetted by SAM, HA, and MA to ensure accurate interpretation, and the broader team reviewed the findings for further discussion.

## Results

### Overview of Findings

**Figure 2.**
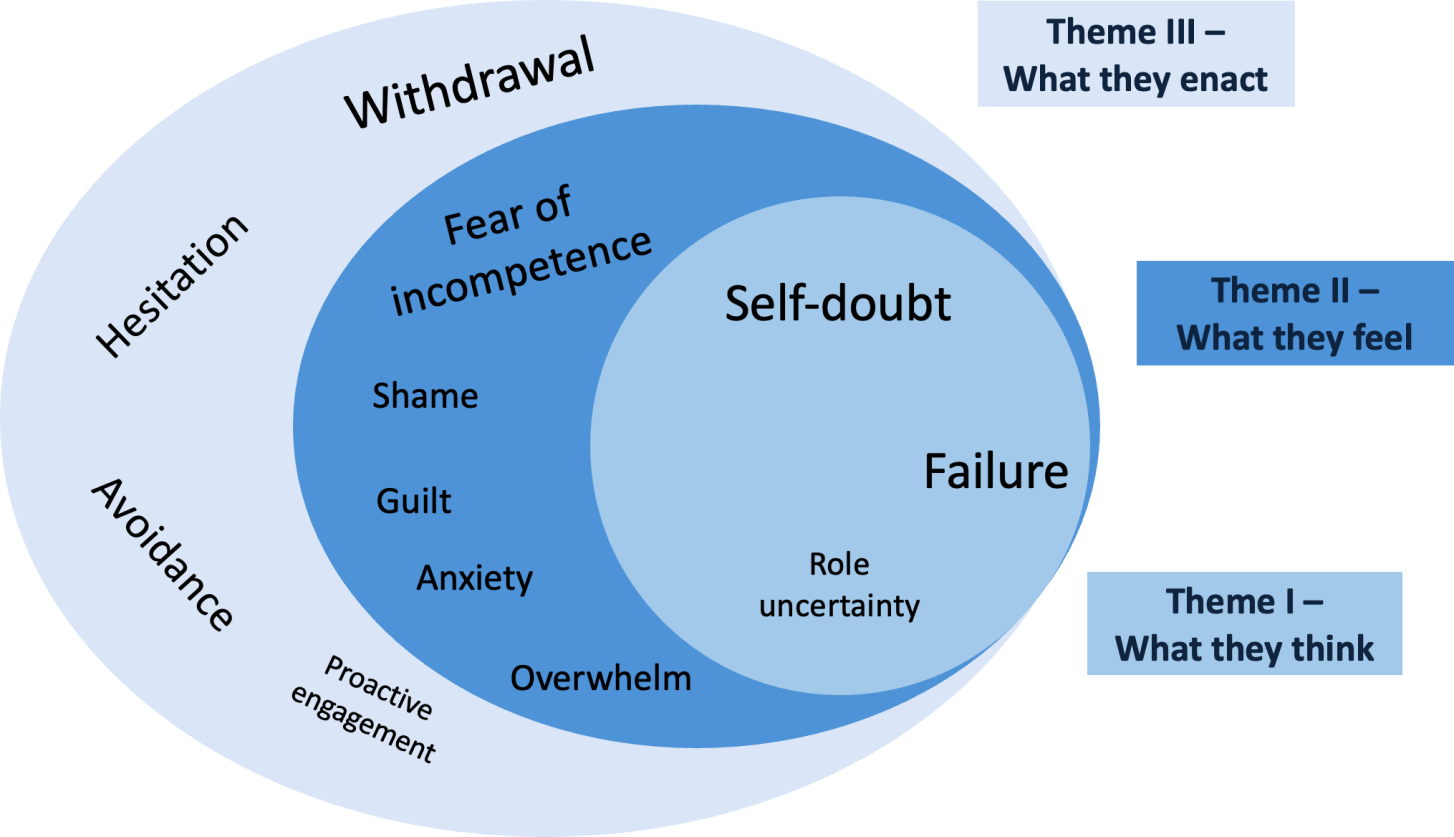
Three interconnected themes: thinking, feeling, and enacting. These themes illustrate the dynamic progression of professional identity formation. Subthemes are embedded within and sized proportionally to reflect their relative frequency and prominence in the data.

Through QD methodology, we adopted a factist perspective for the content thematic analysis [15,16] of the 26 reflective essays. We produced an unadorned description and comprehensive summary of the phenomenon of interest using low inferential stance of participants’ language. This analysis revealed how year 4 medical students think, feel, and act during their first clinical placement in GP. Guided by Lave and Wenger’s [5] concept of LPP, these themes illustrate how students negotiate their identities at the margins of practice communities and gradually move toward fuller participation. By remaining close to the data and providing a thick description of PIF as an abstract phenomenon, we present our findings as 3 interconnected dimensions of PIF—thinking, feeling, and enacting—which capture the shifts students undergo as they move from peripheral to core participation in clinical practice. The innermost circle represents the internal cognition beginning with self-doubt, expanding into failure and role uncertainty, whereas the middle circle yields the emotional processes students described, ranging from fear of incompetence, guilt, shame, anxiety, and overwhelm. These internal experiences influenced the outward behavioral responses, which are depicted as the outer layer, ranging from withdrawal and avoidance to hesitation and rarely a proactive engagement. The size of the font reflects the frequency of the subtheme expressed across the reflective narratives. These interconnected themes share several common elements, which are illustrated in Figure 2.

Students’ reflections illustrated how cognitive, emotional, and behavioral responses interacted dynamically during challenging encounters, shaping how they made sense of their early clinical experiences. Next, we provide a detailed account of the 3 identified themes supported by the verbatim excerpts from the reflective essays.

### Theme I: What They Think

Thinking involved students making sense of their clinical experiences by reflecting on their existing medical knowledge and its application in actual practice and ethical decision-making, as well as the expectations placed on them in their clinical placements and their role within the wider health care team. These cognitive processes appeared as failure, self-doubt, and role uncertainty, characterized by a lack of more profound awareness of the complexities of patient care.

Students taking patient history, clerking, and performing technical skills on actual patients realized the disconnected realities of classroom versus real-world practice.

*a person’s illness experience is deeply intertwined with his/her thoughts; with seemingly minor procedures like blood withdrawal being capable of causing significant distress when a person is uncomfortable about them.*

A student mentioned that performing phlebotomy for the first time on an actual patient seemed much more complex and challenging despite attempting this procedure in vitro (simulated environments), which led to a sense of failure.

*This patient was obese and I neither saw nor palpated the vein. I was hesitant, removed my gloves, and put the equipment away. I kept overthinking and doubting my abilities. Because of that, I lost confidence in doing other venepunctures*

Similarly, one of the students mentioned that the cultural sensitivity taught during case-scenario analysis and express-team-based learning sessions [17] of personal and PIF did not seem to translate into the correct practices due to the untamed beast of unconscious bias, which hinders effective communication. Stereotyping appeared to be the most common flaw because of patients’ physical attire and appearances.

*I was wrong about this patient, but worse, I used a stereotype and approached the consult with prejudice. I latched onto a stereotype and minimized her concern so quickly. I used an anchoring bias to make my judgment, which was rooted more in prejudice and arrogance than evidence- based medicine.*

Students from an external or foreign culture in the clinical setting especially confessed to jumping to conclusions due to false perceptions of gender-based restrictions, language barriers, and conservative cultural norms in the contextual clinical settings.

*I must reassess my attitudes towards discussing sex and identify unrecognized barriers hindering me from effective communication in this aspect. Becoming more confident in my knowledge on the subject (culture) would assist me in navigating future situation. I recognized the need for diligent self-awareness of my implicit biases and in developing my cultural humility.*

Another thought process worth highlighting is the lack of clear expectations of roles and responsibilities in terms of to what extent they can have thoughtful discussions with the patients, disclosure about their prognosis and treatment, and unfamiliarity with the scenarios surrounding confidentiality, informed consent, and patient autonomy. This cognitive dissonance often triggered cognitive conflict that can perpetuate uncertainty precipitating self-doubt and hesitation.

*I was uncomfortable and unsure of what to say in the absence of my GP Tutor. I was afraid of saying the wrong thing and falsely reassuring the patient. Being young and unmarried I felt in-equipped to navigate such a complex situation.*

In the next theme, we will see how these cognitive realizations actually translate into “feeling or emotive” aspect of PIF.

### Theme II: What They Feel

Feelings are the emotional perceptions of the thought processes that we described in our first theme, triggered by uncertainty, challenges, and expectations about students’ roles and a fractured translation of knowledge to practice. Fear, guilt, shame, anxiety, and sense of overwhelm were the four dominating feelings we encountered in those 26 reflective essays.

Fear was the most encountered feeling, and it appeared in many varied situations. Students feared “harming the patient,” “making mistakes,” “challenging hierarchical decisions,” and “false reassurances to the patient.” These fears of inadequacy reflect a significant mental toll, which can hamper the behavioral capacity of students, either leading to avoidance, hesitancy, and withdrawal from such crucial learning opportunities. We have supported these findings by the following excerpts, respectively.

*I became scared when I struggled to remove the first tube, I was afraid that I would accidentally move the needle too deep and puncture the vein. When the lab technician realized my needle had come out, I felt guilty that the patient would have to be punctured again on the other side because my job was not good enough.*

Self-doubt and making mistakes stemmed from feelings of inadequacy when thrust into unfamiliar scenarios without structured preparation. Making students fear the subpar consequences in unfamiliar situations like taking a history from a pediatric patient.

*I thought that I would be wasting the parents’ time if I didn’t get straight to the point, so I barely gave myself a chance to compose myself. I ended up rushing the encounter to keep up with my racing thoughts. During that time, I was constantly worrying about whether I was making mistakes or embarrassing myself. I unintentionally overlooked fundamentals of communication skills like eye contact as a consequence. Unfortunately, I experienced a feeling of inadequacy as a result of the encounter and my lack of knowledge in that area.*

However, there were few voices who felt that to overcome fear, they must try.

*I believe now that to overcome a fear, one must jump into the deep end. I find that through this experience, I learned that it is perfectly fine to take a step back and ask for help when necessary.*

The second common emotion is “guilt and shame” when students could not meet self-imposed and patients’ expectations of professional standards while doing a simple procedure of phlebotomy.

*I felt ashamed that I was not able to perform under pressure, and I was embarrassed that this happened in front of the patient.*

Another student’s narration of a “well-child visit” encounter depicts the moral trauma felt by the student, which was shocking to begin with, but then led to an extreme feeling of shame, where students are not equipped to correct seniors or raise concerns in a hierarchical setting of medical practice.

*I think I was in shock. I had just witnessed a child’s legs forced open, by 3 adults, without explanation, permission, or communication. I had just witnessed a child scream and kick in fear and lack of understanding as adults she trusted forced her legs open. There must be a better way. I feel I participated in a traumatic event for the child, one that might stay with her unconscious, if not a developed memory. I feel at a loss on how to discuss this with the doctor. I feel (ac)complic(e) in causing unnecessary trauma to a child. There needs to be a better way, a more human way.*

Guilt emerged from the imposter syndrome, where students felt incompetent, “unable to perform a given task,” and felt responsible for distressing the patient.

*I felt guilty because I stereotyped and minimized her concern. I don’t like to think I’m prejudice, but I was prejudiced toward her. I boxed her into a stereotype that may not even be real.*

Surprisingly, guilt seemed to prompt students to take self-assessment and a desire to improve and learn better. On the contrary, shame drifted them toward avoidance and hesitancy. And this feeling of guilt was perpetuated when the health care staff showed their displeasure, even implicitly.

*I froze but the phlebotomist handled it and she expressed her anger with her body language, after a while I was standing in the booth and a colleague of hers came in and they spoke in their native language which I understood but she didn’t know that I could understand them, she told her colleague that I was useless and I was going to be a failure because I couldn’t perform a simple phlebotomy, she said a lot of hurtful things about me but I said nothing and stood there and once I got home I started doubting myself and cried about it because I thought I was worthless and not a good medical student and will be a failure.*

Next, the feeling of anxiety and overwhelm was mainly tied to the procedural skills and communication elements where students required independent decision-making and interpretation. One student described paralyzing anxiety while measuring blood pressure incorrectly, which led to avoidance of the procedure afterwards.

*Failing to measure patient’s BP correctly resulted in range of negative emotions and in confidence which led me to avoid measuring the BP of other patients rather than accepting my mistake and work on improving my skill.*

Another student felt extremely overwhelmed by the chaos of medical practice—the noise, busy scenes, medical equipment, and the emotions of worried families. The distress of patient relatives spread like an emotional contagion, affecting the student’s mental and emotional well-being.

*I experienced an unforgettable moment one Thursday evening when I heard screams coming from the corridors of the emergency department. I immediately ran out to investigate and found a chaotic scene with a mother carrying a bleeding 4-year-old boy, accompanied by around 15 friends who were all crying and screaming. I was scared, anxious, and worried about the child’s condition. I was also overwhelmed by the chaos and noise in the health care center.*

In the next theme, we will see how these emotions translate into the affective component of “enacting” and tell which direction these narratives took in terms of remaining stuck to LLP or progressing toward the core aspect of communities of practice.

### Theme III: What They Enact

Enacting is the translation of thoughts and emotions into observable behaviors, which ranged from being proactive to complete avoidance. Considering these reflective essays provide a snapshot of students’ clinical experiences, this theme shows a mix of *hesitation, participation,* and *some moments of growth*.

Patient care served as a catalyst to reveal the subtleties and nuances of students’ actions. The majority of the voices reflected low efficacy in the form of underconfidence in performing technical skills, which led them to hesitation and withdrawal. Few voices were heard who felt confident and approached the clinical behaviors effectively. Softer skills like communication, rapport building, and gaining patients’ trust were the trickiest.

A prominent voice of incompetence emerged in dealing with ethical challenges. Maintaining probity and truthfulness in the middle of practicing and learning skills seemed the most challenging.

*As the nurse and I walked around the station, we came across a patient who needed an injection. I agreed to the nurse when he asked if I wanted to give an IM injection. However, before I could ask the patient for consent to administer the injection, the nurse informed him that I was a doctor and that I would be administering the injection. This surprised me because obtaining consent for any medical procedure was standard practice. I tried to tell the patient, but I couldn’t, so I gave the injection.*

Students learned that effective communication was key to patient care, especially in sensitive situations where they struggled with breaking bad news, realizing later that staying composed and exhibiting just the right amount of empathy was as important as delivering medical facts.

*I need to learn how to communicate bad news in a sensitive and empathetic manner without inappropriate emotional display and how to provide support to patients during emotionally difficult times.*

Nearly half of the essays showed a similar trend toward technical skills, with only one-third able to perform these skills competently. And this lack of self-efficacy was also attributed to patients’ perceptions and times of COVID-19.

*I really wanted to practice but most patients didn’t give me the chance to practice on them and they were underestimating the new generation of medical students as they believe that we are not good enough. Some of them gave negative comments, they said that they will never trust covid medical students batch, as we were in a blinded learning for almost 2 years. Such comments made me question my abilities and made me full of self- doubt myself because I’ve heard the same comment from a lot of patients.*

One of the students mentioned that a major boost to their self-efficacy was external. Attending and supervising health care staff were the significant contributors to that.

*After removing the first skin tag my GP whispered “good job” and I was able to remove six more with confidence. I felt a sense of accomplishment that was indescribable, and I thanked my GP for helping me and giving me a chance to do this procedure.*

Many student narratives described initial reluctance to participate, often due to self-doubt or fear of making mistakes. However, some quickly transitioned into more active roles, showing initiative and professional autonomy. A visibly annoyed GP, frustrated by patients repeatedly interrupting examinations, appreciated the students’ proactive efforts to manage the situation.

*I noticed that my doctor was visibly frustrated by this since it was disrupting her consultations and the patients were losing their confidentiality. I asked my doctor to let me handle those patients. The next time this happened, I asked the patient at the door to meet me outside. I started by asking nicely “Why did you open the door?” and the patient replied, “I just need the lab test results and I’ve been waiting for hours now.” After understanding the patient’s intention, I de-escalated the situation by telling that I completely understand his frustration; however, the doctor is under the pressure of having to follow the order of what the system says, it would break the patient’s privacy, and that once his turn comes in, I’ll make sure to personally call him in.*

## Discussion

### Principal Findings

Students’ reflections reveal a cohort entering the CLE with limited realistic preparation, mirrored by variably prepared GP tutors. Their narratives do not express explicit expectations of seamless transfer but instead reflect prereflective epistemological assumptions—the tacit belief that theoretical knowledge should translate into competent clinical action. When confronted with the realities of practice, students experienced shock and disorientation, signaling insufficient preparation for the well-documented fracture between knowing and doing. Without structured supervisory support to help them process this disjunction, their early experiences were marked by uncertainty, self-doubt, and emotional strain.

These accounts illuminate how students begin to think, feel, and enact as their professional identities take shape [13]. Early transitional moments were characterized by cognitive dissonance, emotional distress, and hesitant behavioral enactment. While reflection can support sense-making during such challenges, it is not sufficient on its own. It must be paired with preparatory activities and supervisory scaffolding to more effectively support students’ PIF and their movement from peripheral to fuller participation in clinical communities of practice. This interpretation aligns with existing literature emphasizing the need for structured scaffolding during the transition from preclinical learning to the CLE. In the following sections, we further situate these findings using Wenger’s LPP framework [5].

### Fracture Between Knowledge and Knowing

A recurring theme was the fracture between theoretical knowledge and its real-world application, underscoring a persistent epistemological gap in medical education. Although students did not explicitly anticipate an effortless transition, many experienced cognitive dissonance when confronted with the uncertainty and practical demands of clinical environments. Their narratives varied in detail but revealed a shared emotional landscape of apprehension, fear, and unpreparedness. These reactions do not reflect naïve expectations but rather insufficient preparation for the well-recognized divide between knowing and doing. This dissonance reflects a deeper epistemic tension: students implicitly equated knowledge acquisition with readiness for action [18].

This assumption was particularly evident in their beliefs about “hard” technical skills, where they expected classroom learning to translate directly into competent performance. When confronted with failures in basic procedures, students who felt prematurely “cloaked in competence” experienced abrupt identity shock, often marked by shame, guilt, anxiety, and declining self-confidence [19,20]. Such experiences stunt LPP and add to the slow pile-up of burnout quotient. Conversely, there was a relatively mature understanding of the epistemological perspective when it came to “soft” skills, like professionalism-related matters of bias, consent, confidentiality, culture, and challenging hierarchy [17]. This can be attributed to the preexisting understanding of professionalism as an abstract concept. Hence, students had internalized the interpretation of professionalism interpretation, which is based on the evaluation of evidence across contexts and experts [21].

The nature of knowing ranged from simplicity for technical skills to justification for professionalism matters [21]. In the context of medical education, there is a perception that biomedical knowledge is often easier to acquire in an ideal environment compared to the biopsychosocial element*—understanding of what it means to be a physician* as mentioned by Whitcomb [22]. And that’s exactly what our students understood. However, when it came to execution, they were struck by an identity shock, which had significant repercussions on their ability to learn, perform, and grow. Considering that most medical schools are still operating within the positivist paradigmatic orientations of their curricular structures, such results are expected [10]. To navigate that, introducing learning activities that increase the students’ understanding of the CLE and the uncertainty, sense of incompetence, and failures that are associated with practicing medicine should be introduced [23]. An effective implementation of assessment methods that reflect epistemological complexity, such as self-reflective narratives and longitudinal evaluations, combined with early clinical exposure, is key. This gradual familiarization with the ambiguity, messiness, and relational complexity of real-world clinical practice can help students become more resilient to the uncertainty that awaits them.

### Emotional Labor

The tension between expectations and real-world clinical failures is likely to result from the complex and contextualized patient encounters that constitute clinical medicine. The slow unveiling of the complexity inherent in the simple task of measuring blood pressure and phlebotomy seemed a shocking reality for the students. Performing such skills on a human patient versus a simulator is much more complex than anticipated and triggers the destabilizing emotion of shame and guilt [20]. Only very few experienced health care providers were able to recognize a student’s struggle with such emotional responses, missing an excellent opportunity to support them. Another source of stress emerged from the hidden curriculum [24] “little white lies” [25] when the nurse introduced the student as a doctor to practice IM injection. Students felt ashamed, amalgamated with fear of consequences, and were unable to challenge the attending who performed intimate examinations without proper consent and exposure.

Another source of emotional labor emerged from the materiality of everyday life in a CLE. Emotionally charged patient families, chaotic and loud environment buzzing with sounds of technology, and material things have been reported to hinder the learning process [26]. Fenwick’s [27] seminal work has highlighted the limited attention to materiality (objects, technologies, and their nature) in learning. Medical curricula focus too heavily on human-centered practice, ignoring the vital role of material factors like technology and environment in shaping clinical learning. Students struggle with the tension between standardized medical protocols and the unpredictable realities of patient care, leading to anxiety and uncertainty. Without structured support mechanisms to process emotional distress, students may struggle to engage confidently in clinical settings, reinforcing avoidance behaviors that limit their participation and professional growth [20]. This advocates for the current apprenticeship approach to training to include a developmental perspective, providing effective feedback and supporting learner self‐assessment and reflection. Facilitating reflective debriefings after stressful experiences and acknowledging the presence of shame and guilt in the learner by avoiding humiliation and leveraging effective feedback can provide students with tools to navigate these complex emotions constructively [19].

### Agency Versus Avoidance

The reflections endorsed our recent work [28] that a supportive attending and superior were decisive factors in boosting medical students’ self-efficacy. Judging medical students and setting harsh, unrealistic expectations of being “cloaked in competence” are the most dreadful fears students perceive [29,30]. Learning happens when psychological safety is assured. Indeed, published literature suggests that supportive and encouraging preceptors who discuss failures [31] and the perplexity of CLE and provide constructive feedback can lead to *prepared* graduates [9]. Unlike Wald et al [9], none of the reflections in our case mentioned any discussion of mistakes, identification of areas of improvement, or debriefing sessions aimed at maximizing learning [32]. This is a critical omission, highlighting the need for educators to create spaces within the curriculum to discuss “failures” with students, preceptors, and clinical teachers [11]. According to Wenger’s LPP, constructive failure processing mechanisms should be supported through sophisticated reflection-in-action [33]. This process should encompass awareness, collaboration, negotiation, evaluation, and realization to foster a conscious understanding of the transformation occurring in our medical students. Such structured processes will lead to graduated autonomy and avoidance of withdrawal. These trajectories of support, reflection, and graduated autonomy mirror the broader identity formation processes described earlier, where learners move from peripheral participation toward fuller membership in professional communities [5]. This leads us to consider the broader educational implications of our findings.

### Educational Implications

Considering medicine is a social science and students perform in the social contexts of clinical environments, we need to introduce more constructionist nonbinary perspectives to the biomedical elements of the curriculum by providing more self-directed study opportunities [34]. Our findings expose structural and pedagogical limitations. Students thrown into the CLE without adequate psychological preparation and a structured supervisory support will be startled by the practical, emotional, and epistemological complexities inherent in the context. To move beyond that, medical curricula must frame uncertainty and failure as aspects of early clinical participation. Senior peers and role models sharing their reflective narratives of failure and fear provide psychological assurance and make failure, multiple failures, and success part of the whole process [35]. However, training the clinical supervisors and their ability to support, debrief, and role-model are essential. Within this broader scaffolding, reflective practice should be positioned not as a cure-all but as a developmental tool that complements preparatory and supervisory strategies. Despite the known benefits of reflective practice in the medical curriculum, we are not yet reaping the full benefits [36]. The literature suggests that we need to reflect upon reflection [10]. Incorporating uncertainty curricula [23], dealing with failures to normalize struggle [37], graduating autonomy models [38], and constructing inclusive, nonjudgmental learning spaces punctuated with frequent reflections upon reflections are required. Early identity struggles, if left unaddressed, may carry over into residency, where clinical demands intensify, increasing the risk for emotional exhaustion and disengagement [39]. By implementing structured identity support and normalizing professional uncertainty early in training, medical schools can better prepare students for the emotional and professional complexities of long-term clinical practice.

Reflective essay accounts rooted in the discipline of psychology have nicely elucidated these problems evident from our results. However, the application of reflection is heavily steered by the assessment discourse and is divorced from original theories of reflection and reflective practice [40]. So clearly, the solution lies one step ahead—postreflective writing group reading/listening/discussions [8,41] and in multidisciplinary domains [42]. Inspired by Hodges [40], we advocate for ongoing, theory-informed exploration of reflection in medical education, particularly its role in navigating complexity, challenging dominant discourses, and shaping knowledge through diverse epistemological lenses and predominantly a critical social inquiry. Yet, the solution of viewing medicine from a more sociological perspective is compelling; the medical profession strongly believes in identity formation, whose disciplinary roots are entrenched in philosophy, psychology, and sociology. New ways of knowing in medical schools gravitate toward psychology alone [43]. However, the results of this study point to a more holistic approach when teaching professionalization to medical students in this humane profession [44]. We must look and discuss with our students the elements of resilience, humility, uncertainty, accepting failures, and limitations rooted in psychological and sociological principles [11]. Such multidisciplinary understanding may bridge the disconnect between the nature of knowledge and the nature of knowing. Table 1 provides guidance for curricular modifications to address the cognitive, emotional, and behavioral challenges identified in students’ reflections.

**Table 1.** Summary of the 3 thematic domains (thinking, feeling, and enacting) derived from students’ reflective accounts, with proposed key educational implications for curriculum design to support the process of professional identity formation (PIF).

Theme	Core insights from student reflections	Educational implications for curriculum design and PIF support
I. Thinking: cognitive dimension	Students experienced a fracture between theoretical knowledge and real-world application, revealing naïve epistemological beliefs that knowledge automatically translates into competence. They expressed uncertainty about their roles, ethical decision-making, and navigating culturally complex interactions.	Introduce early, structured clinical exposure with clear role expectations; provide orientation to epistemic uncertainty; embed narratives from senior peers and case-based discussions that highlight real-world complexity before clinical placements; incorporate shared reflective circles after the clinical placements to make epistemological tensions explicit and discuss uncertainties communally.
II. Feeling: emotional dimension	Students reported intense fear, guilt, shame, anxiety, and overwhelm, particularly during procedural tasks, culturally sensitive interactions, and ethically ambiguous situations. Emotional distress often stemmed from lack of preparation and insufficient support structures.	Implement preplacement skills practice on peers and simulations; create psychologically safe learning environments; integrate structured debriefings and shared reflective circles to process emotional labor, acknowledge shame/guilt as part of professional growth, and collectively normalize struggle both earlier in the clinical placements and the end of clinical placements
III. Enacting: behavioral dimension	Students’ actions ranged from avoidance and withdrawal to proactive participation, shaped strongly by supervisory style and perceived psychological safety. Supportive preceptors enhanced agency; lack of feedback led to hesitation.	Train general practice tutors in feedback, debriefing, and psychological safety strategies; integrate shared reflection and postreflective dialogue sessions to support students in articulating their experiences, reframing failures, and sustaining engagement in clinical communities.

### Limitations and Strengths

This study offers insights from a Middle Eastern CLE, a context that is underrepresented in the literature on PIF, thereby contributing to the global understanding of medical education. The QD method in this study focused on reflective essays because they provided us with a unique window into critical moments that prompt students to engage in introspection. By providing vivid personal accounts, these essays highlighted the students’ narratives of key learning moments and challenges. Also, having student researchers from the same cohort helped us to interpret the data more truthfully. Their shared contextual understanding enabled us to ensure that the analysis remained authentic and reflective of the lived experiences of their peers, reducing the likelihood of misinterpretation and enhancing the credibility of the findings.

However, it must be acknowledged that this research has limitations. This cross-sectional analysis of one subset of a single cohort at a European medical school in a Middle Eastern context might be a naïve effort, limiting contextualization of our research findings. We analyzed 26 reflective essay accounts of students focused solely on GP placements, and the findings may not fully capture the professional identity struggles that occur in other specialties, which may have different levels of complexity. Moreover, we captured students’ narrations, using reflective essay accounts, which can be merely the medium for expressing inner idealistic thoughts and feelings (when writing reflections or participating in discussions) [11] and are not drawn from discourse analysis [45]. Additionally, these reflective essays were submitted as part of an assessment process, and the findings may be subject to social desirability bias, where students may downplay certain struggles or amplify professionalism, knowing their work is being evaluated. Further, because students were prompted to describe “challenging or difficult encounters,” their reflections may be disproportionately weighted toward negative experiences. This instruction may have introduced a response bias that limits the representation of more balanced or positive interactions. We also acknowledge that, as with any single research method, it is not a holistic attempt, and we may find alternative aspects with other observational, ethnographic, or phenomenological examinations. Despite this, we believe this work reminds us of the complexities of transitioning moments in medical students’ PIF.

### Conclusion

We have presented the findings of medical students’ transitioning moments from preclinical learning to the CLE. These reflective essays showed that students entered clinical practice with little to no realistic preparation and nonexistent structured supervisory support. The fluid and complex nature of PIF meant that this lack of preparedness inevitably gave rise to cognitive dissonance, emotional distress, and hesitant enactment. Students’ relatively simplistic prereflective epistemological beliefs contributed to the cognitive dissonance, precipitating emotions of shame and guilt, which led to avoidance and withdrawal. Recognizing these pedagogical gaps is essential. Medical curricula and pedagogical approaches must equip students to manage the expectations, complexities, and uncertainties of the CLE before entry. This can be achieved through early skills training, opportunities for peer practice, exposure to senior peers’ narratives, and clarity regarding roles and norms. However, such efforts cannot operate in isolation. Equipping GP tutors with strategies to support, debrief, and guide learners through these crucial moments is equally vital. Within this broader scaffolding, reflection remains a critical tool—not a cure-all but a complementary mechanism for sense-making, growth, and resilience. By integrating structured preparatory activities with theory-informed communal reflective circles, we can help these anxious members of the team navigate uncertainty, normalize failure, and cultivate resilience. As educators, we have already bridged the gap between knowledge and knowing by acknowledging the profession’s demand of an equal mastery of scientific expertise alongside humanistic engagement. However, small final steps are needed. We need to reflect upon reflections. Let us make such curricular spaces to support the PIF of our new clinical team members.

## Abbreviations

CLE: clinical learning environment
GP: general practice
LPP: legitimate peripheral participation
PIF: professional identity formation
QD: qualitative descriptive
RCSI-MUB: Royal College of Surgeons in Ireland – Medical University of Bahrain

## References

[1] Cruess RL, Cruess SR, Boudreau JD, Snell L, Steinert Y (2014). Reframing medical education to support professional identity formation. Acad Med.

[2] Nordquist J, Hall J, Caverzagie K (2019). The clinical learning environment. Med Teach.

[3] Artemeva N, Rachul C, O’Brien B, Varpio L (2017). Situated learning in medical education. Acad Med.

[4] Irby D Improving environments for learning in the health professions. https://macyfoundation.org/assets/reports/publications/macy_monograph_2018_webfile.pdf.

[5] Lave J, Wenger E (1991). Situated Learning: Legitimate Peripheral Participation.

[6] Gaufberg EH, Batalden M, Sands R, Bell SK (2010). The hidden curriculum: what can we learn from third-year medical student narrative reflections?. Acad Med.

[7] Wald HS (2015). Professional identity (trans)formation in medical education: reflection, relationship, resilience. Acad Med.

[8] Shapiro J, Kasman D, Shafer A (2006). Words and wards: a model of reflective writing and its uses in medical education. J Med Humanit.

[9] Wald HS, White J, Reis SP, Esquibel AY, Anthony D (2019). Grappling with complexity: medical students’ reflective writings about challenging patient encounters as a window into professional identity formation. Med Teach.

[10] Mann K, Gordon J, MacLeod A (2009). Reflection and reflective practice in health professions education: a systematic review. Adv Health Sci Educ Theory Pract.

[11] Benbassat J (2013). Undesirable features of the medical learning environment: a narrative review of the literature. Adv Health Sci Educ Theory Pract.

[12] Sandelowski M (2010). What’s in a name? Qualitative description revisited. Res Nurs Health.

[13] Gibbs G (1988). Learning by Doing: A Guide to Teaching and Learning Methods.

[14] Branch WT (2005). Use of critical incident reports in medical education. A perspective. J Gen Intern Med.

[15] Kim H, Sefcik JS, Bradway C (2017). Characteristics of qualitative descriptive studies: a systematic review. Res Nurs Health.

[16] Guraya SS, Guraya SY, Harkin DW, Ryan Á, Mat Nor MZB, Yusoff MSB (2021). Medical Education e-Professionalism (MEeP) framework; from conception to development. Med Educ Online.

[17] Guraya SS, Guraya SY, Doubell FR (2023). Understanding medical professionalism using express team-based learning; a qualitative case-based study. Med Educ Online.

[18] Evans RG (2003). Patient centred medicine: reason, emotion, and human spirit? Some philosophical reflections on being with patients. Med Humanit.

[19] Bynum WE, Goodie JL (2014). Shame, guilt, and the medical learner: ignored connections and why we should care. Med Educ.

[20] Bynum WE, Teunissen PW, Varpio L (2021). In the “shadow of shame”: a phenomenological exploration of the nature of shame experiences in medical students. Acad Med.

[21] Knight LV, Mattick K (2006). “When I first came here, I thought medicine was black and white”: making sense of medical students’ ways of knowing. Soc Sci Med.

[22] Whitcomb ME (2006). The teaching of basic sciences in medical schools. Acad Med.

[23] Kitto SC, Chesters JE, Villanueva EV, Fox JG (2004). Normalising uncertainty in undergraduate clinical transition seminars. Focus Health Prof Educ.

[24] Guraya SS, Kearney GP, Doyle F (2024). “Busting the hidden curriculum” a realist and innovative perspective to foster professional behaviors. Front Med.

[25] Lingard L, Schryer C, Garwood K, Spafford M (2003). “Talking the talk”: school and workplace genre tension in clerkship case presentations. Med Educ.

[26] Goldman E, Plack M, Roche C, Smith J, Turley C (2009). Learning in a chaotic environment. J Workplace Learn.

[27] Fenwick T (2014). Sociomateriality in medical practice and learning: attuning to what matters. Med Educ.

[28] Habib H, Niinuma SA, Alrefaie K (2025). Shades of acceptance and adjustment; a discursive psychological analysis to showcase empathy in medical students. BMC Med Educ.

[29] Jin CJ, Martimianakis MA, Kitto S, Moulton CAE (2012). Pressures to “measure up” in surgery: managing your image and managing your patient. Ann Surg.

[30] Kitto S, Alexanian J, Goldman J (2022). Researching Medical Education.

[31] Kroll L, Singleton A, Collier J, Rees Jones I (2008). Learning not to take it seriously: junior doctors’ accounts of error. Med Educ.

[32] Branch WT, Paranjape A (2002). Feedback and reflection: teaching methods for clinical settings. Acad Med.

[33] Orsmond P, McMillan H, Zvauya R (2022). It’s how we practice that matters: professional identity formation and legitimate peripheral participation in medical students: a qualitative study. BMC Med Educ.

[34] Frambach JM, Driessen EW, Chan LC, van der Vleuten CPM (2012). Rethinking the globalisation of problem-based learning: how culture challenges self-directed learning. Med Educ.

[35] Nishigori H, Shimazono Y, Busari J, Dornan T (2024). Exploring yarigai: the meaning of working as a physician in teaching medical professionalism. Med Teach.

[36] Wald HS, Reis SP (2010). Beyond the margins: reflective writing and development of reflective capacity in medical education. J Gen Intern Med.

[37] Klasen JM, Lingard LA (2019). Allowing failure for educational purposes in postgraduate clinical training: a narrative review. Med Teach.

[38] Ramani S, Leinster S (2008). Amee Guide no. 34: teaching in the clinical environment. Med Teach.

[39] Peterson WJ, House JB, Sozener CB, Santen SA (2018). Understanding the struggles to be a medical provider: view through medical student essays. J Emerg Med.

[40] Ng SL, Kinsella EA, Friesen F, Hodges B (2015). Reclaiming a theoretical orientation to reflection in medical education research: a critical narrative review. Med Educ.

[41] Lie D, Shapiro J, Cohn F, Najm W (2010). Reflective practice enriches clerkship students’ cross-cultural experiences. J Gen Intern Med.

[42] Gerrity MS, Earp JAL, DeVellis RF, Light DW (1992). Uncertainty and professional work: perceptions of physicians in clinical practice. Am J Sociol.

[43] Roberts J (2006). Limits to communities of practice. J Manag Stud.

[44] Amin A, Roberts J (2008). Knowing in action: beyond communities of practice. Res Policy.

[45] Taylor C (2006). Narrating significant experience: reflective accounts and the production of (self) knowledge. Br J Soc Work.

